# Revisiting Machine Learning Potentials for Silicate Glasses: The Missing Role of Dispersion Interactions

**DOI:** 10.1021/acs.jctc.5c00218

**Published:** 2025-04-24

**Authors:** Alfonso Pedone, Marco Bertani, Matilde Benassi

**Affiliations:** Department of Chemical and Geological Sciences, University of Modena and Reggio Emilia, Modena 41125, Italy

## Abstract

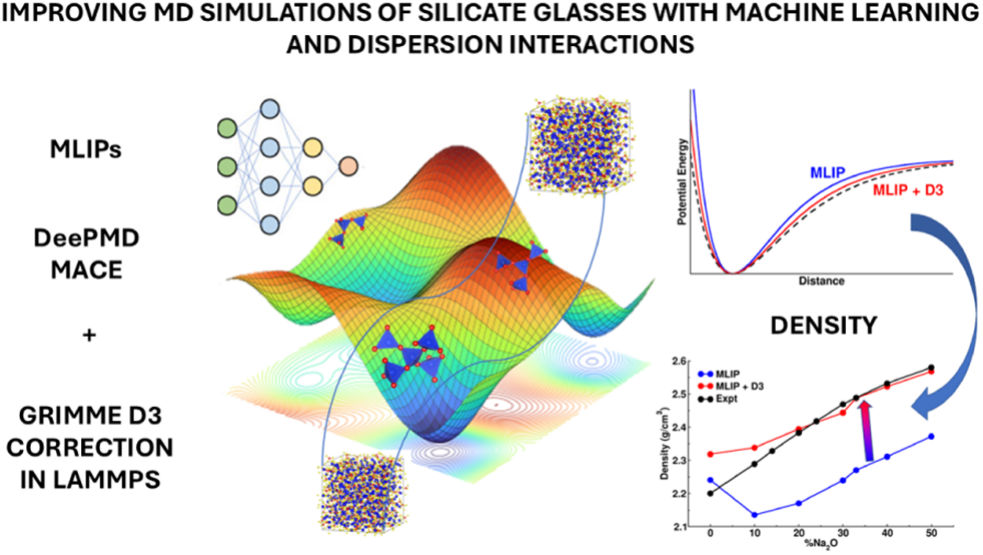

Machine learning interatomic potentials (MLIPs) offer a promising alternative to traditional force fields and ab initio methods for simulating complex materials such as oxide glasses. In this work, we present the first evaluation of the pretrained MACE (Multi-ACE) model [D.P. Kovács et al., J. Chem. Phys. 159(2023), 044118] for silicate glasses, using sodium silicates as a test case. We compare its performance with a DeePMD-based MLIP specifically trained on sodium silicate compositions [M. Bertani et al., J. Chem. Theory Comput. 20(2024), 1358–1370] and assess their accuracy in reproducing structural and dynamical properties. Additionally, we investigate the role of dispersion interactions by incorporating the D3(BJ) correction in both models. Our results show that while MACE accurately reproduces neutron structure factors, pair distribution functions, and Si[Q^n^] speciation, it performs slightly worst for elastic properties calculations. However, it is suitable for the simulations of sodium silicate glasses. The inclusion of dispersion interactions significantly improves the reproduction of density and elastic properties for both MLIPs, highlighting their critical role in glass modeling. These findings provide insight into the transferability of general MLIPs to disordered systems and emphasize the need for dispersion-aware training data sets in developing accurate force fields for oxide glasses.

## Introduction

Silicate glasses are ubiquitous materials used in a wide range of applications, from construction and optics to high-tech electronics and communications.^1,2^ Their utility arises from their unique ability to be chemically tailored for specific properties, making them ideal for numerous industrial and technological fields. Unlike crystalline materials, silicate glasses lack long-range atomic order, making their structural complexity and property prediction a challenge. Understanding how glass composition affects its structure and properties is crucial for designing materials with targeted performance.^3^ However, due to the vast range of possible compositions and the lack of well-defined atomic arrangements, theoretical and computational methods are often required to complement experimental studies.^4,5^

Among computational methods, molecular dynamics (MD) simulations have been instrumental in exploring the atomic-scale properties of glasses.^6−8^ Traditionally, empirical force fields (FFs) have been used to model atomic interactions in silicate glasses.^9−11^ These FFs offer computational efficiency, allowing the simulation of large systems over long timescales. However, they are often limited in accuracy and transferability, particularly when applied to complex or varied glass compositions. The functional forms of these potentials, typically based on two- or three-body interactions, fail to fully capture the subtle effects of the glass’s disordered structure.

In contrast, ab initio molecular dynamics (AIMD) simulations, which compute atomic forces using density functional theory (DFT), offer far greater accuracy. AIMD can account for the electronic structure of the material, providing highly reliable results for atomic interactions. However, AIMD’s computational cost restricts its use to small system sizes (a few hundred atoms) and short timescales (tens of picoseconds), limiting its application to large, complex glass systems or long time scale phenomena.^12−15^

To bridge the gap between the accuracy of AIMD and the efficiency of empirical FFs, machine learning interatomic potentials (MLIPs) have emerged as a powerful alternative.^16−18^ MLIPs aim to reproduce DFT-level accuracy while maintaining computational costs comparable to empirical force fields, enabling the simulation of larger systems and longer timescales without sacrificing precision. One of the most successful MLIP frameworks is Deep Potential Molecular Dynamics (DeePMD), which uses neural networks trained on DFT-calculated data to capture atomic interactions with high fidelity.^19−21^ In our previous work, we developed a DeePMD-based ML potential for sodium silicate glasses, leveraging a training data set that included both low-temperature glass structures and high-temperature melt configurations.^22^ This comprehensive training allowed DeePMD to accurately model a wide range of compositions and temperatures, from crystals to melts and glassy phases, a critical requirement for simulating the glass formation process.

Recently, a message-passing neural network potential based on the Atomic Cluster Expansion (ACE)^23^ descriptor called MACE (Multi-ACE) has been developed and pretrained for 89 elements,^24^ showing great promise for the simulation of materials,^25,26^ including silicate glasses; however, its performance has not yet been fully validated in these systems.

In this study, we focus on sodium silicate glasses, a subset of the broader silicate glass family, to compare the performance of recently developed MACE potential with our specific DeePMD potentials as well as DFT and experimental data. Sodium silicates offer a compositionally simpler system compared to other silicate glasses, and there is a wealth of experimental data available for comparison.

A key difference between DeePMD and MACE lies in their training data. Whereas our DeePMD potential was explicitly trained on both glassy and melt configurations, MACE’s training set does not include high-temperature melts or disordered glass structures. Glasses form through the rapid cooling of melts, and the ability to accurately capture both the melt and the glass phases is essential for realistic simulations. Given that MLIPs are generally not extrapolative, the absence of melt structures in the MACE training set may affect its ability to model glass formation and predict the properties of sodium silicate glasses. Both the pretrained MACE and our DeePMD MLIPs are trained on DFT data coming from PBE functional^27^ which is known to underestimate the dispersion interaction^28^ that was not included into the DFT calculation. This could be the cause of the underestimation of the density observed in previous works from us^22^ and other groups.^29,30^

Therefore, in this work, we perform molecular dynamics simulations of binary sodium silicate glasses (compositions ranging from pure SiO_2_ to 50% Na_2_O) using both our DeePMD and the pretrained MACE model potentials also including the D3 dispersion correction proposed by Grimme^31^ with the Becke–Johnson dumping function^32^ which was implemented in LAMMPS^33^ through an homemade C++ model that we have called D3BJ. We evaluate their performance in reproducing density and key structural properties such as the neutron structure factors, pair distribution functions (PDFs), bond angle distributions, and Q^n^ species distribution, as well as dynamic and mechanical properties like the vibrational density of states (VDOS), the bulk and Young’s moduli and Poisson ratio. By comparing these results with experimental data, we aim to determine whether the pretrained MACE model can reliably generalize to glassy systems, despite being trained solely on crystalline structures and the effect of dispersion correction in both ML models.

This study not only provides a detailed comparison between two advanced machine learning potentials but also highlights the challenges of developing ML models that are both accurate and transferable across a wide range of material systems. If MACE can successfully model sodium silicate glasses, it could become a highly versatile tool for materials science, particularly given its extensive elemental coverage. However, if MACE struggles with the disordered nature of glassy systems, the results will underscore the necessity of including diverse data sets, such as high-temperature melts, in the training process for MLIPs aimed at complex, noncrystalline materials like glasses. Furthermore, the improvement of the structural and dynamical properties potentially given by the explicit inclusion of the dispersion correction can stimulate the debate on the necessity of a fast way to compute it and generate data sets including it directly in the ab initio training data.

## Computational Details

The molecular dynamics simulations with the DeePMD potential were conducted with the LAMMPS software^33^ whereas all the simulations with MACE were performed using the ASE software.^34^

The DeePMD model was trained on a data set of DFT-calculated energies and forces, including both low-temperature glassy and high-temperature melt structures. It used the Se_e3 angular embedding descriptor with a cutoff radius of 7 Å, an embedding network with two hidden layers, and a fitting network with two hidden layers, incorporating the tanh activation function.^22^

As for the MACE potential, we used the MACE-MP-0 model with medium dimension.^25^ MACE’s architecture is designed to capture higher-order atomic interactions through message-passing networks. It utilizes spherical expansion (L_max_ = 3) for local atomic environments, a tensor decomposition channel dimension of 128 channels, and a radial cutoff of 6 Å. The interatomic distance expansion includes 10 Bessel functions fed into a neural network with three hidden layers, using the SiLU activation function.

For both ML potentials, we conducted simulations with and without D3 dispersion correction^31^ with the Becke-Johnson dumping function.^32^ The PyTorch,^35^ implementation was used in ASE for the simulations with the MACE model, while we developed and included in LAMMPS our own C++ module (called D3BJ) for the DeePMD simulations. It is important to highlight that at the time of the simulations carried out for this manuscript the D3 dispersion correction was not included in LAMMPS and the PyTorch implementation was too expensive from the computational point of view. Indeed, several tests performed on a single Nvidia A100 GPU and 24 CPUs on a glass model containing 1500 atoms showed that when using the pretrained MACE model in ASE with the PyTorch-based D3 dispersion correction the simulation rate dropped from 7.16 ps/h without dispersion to 1.72 ps/h with dispersion, making the dispersion-corrected simulation more than four times slower. With our new LAMMPS implementation instead the simulations slow down only of a factor of 1.1.

The sodium silicate glasses were generated using a standard melt-quench procedure.^6^ Initial configurations, with compositions ranging from pure SiO_2_ to 50% Na_2_O, were prepared by randomly placing 1500 atoms in a cubic box and performing classical MD simulations with the PMMCS potential using NVT simulations as described in previous works.^9^ The box volume was adjusted to match experimental densities. Compositions and experimental densities are reported in Table 1.

**Table 1:** Composition and Density of the Simulated Glasses

Glass	%SiO_2_	%Na_2_O	ρ (g/cm^3^)
SiO_2_	100.0	0.0	2.200
NS10	90.0	10.0	2.294
NS20	80.0	20.0	2.385
NS22.5	77.5	22.5	2.385
NS30	70.0	30.0	2.469
NS33	67.0	33.0	2.494
NS40	60.0	40.0	2.550
NS50	50.0	50.0	2.630

The models generated with the PMMCS potential were then heated from 300 to 2500 K at a rate of 5 K/ps, then equilibrated at 2500 K for 50 ps to ensure complete melting with the two MLIPs. Afterward, we quenched the systems to 300 K at the same rate and equilibrated them at 300 K for 100 ps. Simulations were carried out in the NPT ensemble using the Nosé-Hoover thermostat and barostat,^36,37^ with damping and time step parameters set to 25 and 1 fs, respectively.

For the SiO_2_ and NS33 glasses, we computed the VDOS by performing the power spectrum of the velocity autocorrelation functions generated by analyzing a 300K MD trajectory of 50 ps with a time step of 0.1 fs and sampling every 10 fs.

Young’s moduli and Poisson’s ratios were computed by performing molecular dynamics (MD) simulations with strain applied along the x, y, and z directions respectively. For each direction, a series of simulations were conducted to apply incremental strains of 1% up to 3%, using an NPT ensemble where the strained direction was kept fixed while the other two directions were allowed to relax. The strain rate applied was 2·10^–9^ s^–1^. The stress response along the strained direction was recorded at every strain by averaging over multiple MD steps to ensure equilibrium. A linear curve was fitted to the stress–strain data, and Young’s module was obtained by taking the slope of the fitted curve. The Poisson’s ratios were calculated by measuring the lateral strains in the orthogonal directions relative to the applied strain.

For the bulk modulus, hydrostatic compression was simulated by applying isotropic pressures ranging from 0 to 5 GPa by increments of 1 GPa applying a compression rate of 1·10^–13^ s^–1^. An NPT ensemble was used to maintain a constant temperature while the system responded to the pressure variations. The volume of the system was monitored over time, and the bulk modulus was determined by linear fitting to the pressure-volume data, with the modulus calculated as the derivative of the pressure with respect to volume at zero pressure.

## Results and Discussion

### Density

As mentioned in the introduction, previous MD simulations using MLIPs have shown significant errors in predicting glass density.^18,22,29,38^ These discrepancies have often been attributed to the high quenching rates applied in simulations, which differ from the more gradual cooling observed in experiments. However, another potential source of error lies in the accuracy of the ab initio data used to train the MLIPs. The most common DFT approach for calculating the properties of glasses and crystals relies on the PBE functional, which is known to underestimate dispersion interactions.^28^ This underestimation can lead to a corresponding density underprediction when ML potentials are trained on such data.^22,30^

**Figure 1:**
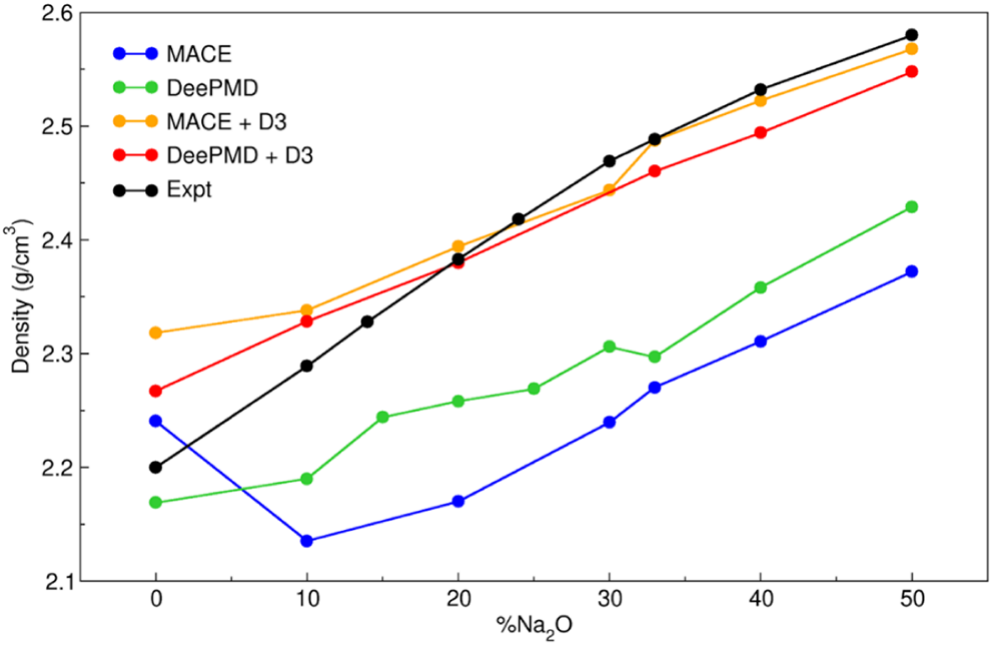
Experimental and simulated density as a function of %Na_2_O.

Figure 1 illustrates the simulated glass density as a function of Na_2_O content, comparing it with experimental data.^39^

The inclusion of the D3 dispersion correction enhances the density reproduction for both MACE and DeePMD MLIPs, emphasizing the significant role of dispersion interactions in accurately modeling the density of glasses. In fact, dispersion interactions dominate over the MLIP model in terms of density reproduction. DeePMD yields better results than MACE without the D3 correction, but this difference diminishes almost entirely once the correction is applied, leaving only a slight overestimation of SiO_2_ density. To quantify the agreement with experimental data, we calculated the percentage Mean Absolute Error (%MAE) for each implementation, which resulted in 7.48% for MACE, 5.43% for DeePMD, 1.42% for MACE-D3, and 1.36% for DeePMD-D3. Overall, we observe that DeePMD-D3 slightly overestimates the density at low Na_2_O content (<20%) and underestimates it at higher Na_2_O concentrations. In contrast, MACE-D3 provides a better reproduction of the glass density at higher sodium oxide content, suggesting a better reproduction of the sodium distribution in the glass. However, the overestimation of glass density at low sodium content, despite the fast quenching rates, suggests that the impact of dispersion interactions (approximately a ∼0.1 g/cm^3^ shift for DeePMD and ∼0.2 g/cm^3^ for MACE) is overestimated.

### Structural Characterization

#### Neutron Structure Factor

**Figure 2:**
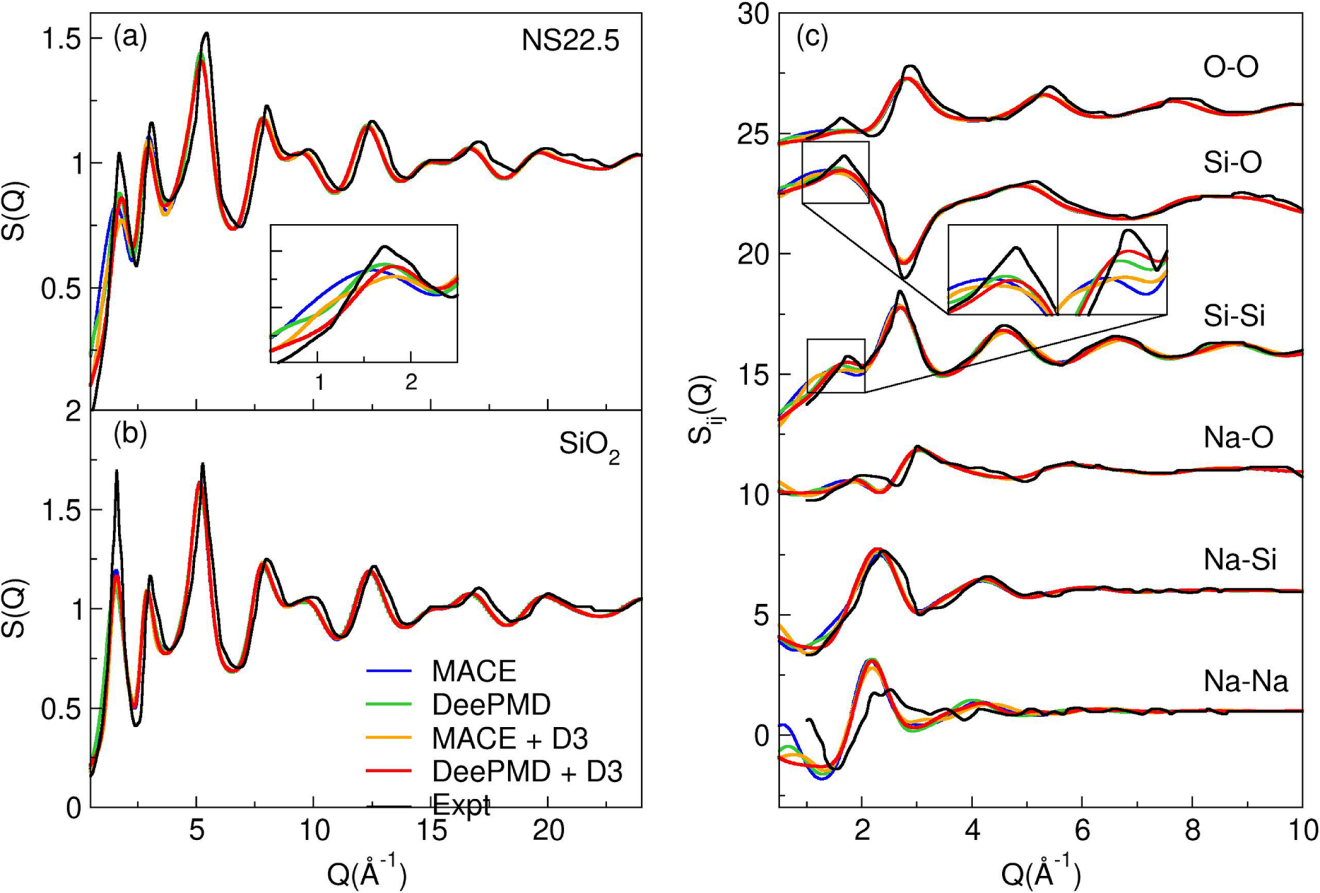
Experimental and simulated neutron total structure factor *S(Q)* of NS22.5 (a) and SiO_2_ (b), and partial structure factor *Sij(Q)* of NS22.5 (c).

Neutron diffraction is a very powerful technique for the investigation of glass structure. In particular, the neutron structure factor (*S(Q)*) gives insight into the medium range order in glasses allowing to separate the contribution of specific couples of elements. Figure 2 shows, in panels (a) and (b), the simulated and experimental^40^ neutron structure factor of NS22.5 and SiO_2_ glasses, respectively. The partial structure factors (*S*_*ij*_*(Q)*) of all the pairs of elements are reported in panel (c).

The total and partial structure factors are generally well reproduced by all the MLIPs, demonstrating their reliability in capturing the structural features of silicate glasses. However, the first diffraction peak (FSDP) of the total structure factor S(Q) for the NS22.5 glass is not accurately reproduced by the MLIPs without dispersion interactions (MACE and DeePMD), as observed in panel (a). In particular the pre-FSDP region shows deviations occurring before the main peak starts to rise. This discrepancy correlates with the underestimation of the glass density and medium-range structural features, particularly the Na-Na distributions and Si-Si distances, as evident in the insets of the partial structure factors *S*_*ij*_*(Q)* shown in panel (c). The box dimensions in the simulations, which are smaller than required for an accurate reproduction of the FSDP, also likely contribute to the discrepancies in the heights of the FSDP.

Notably, the inclusion of dispersion interactions in MLIPs improves the density and medium-range structure, leading to better agreement with experimental data, particularly for the Na-Na and Si-Si correlations. While the pre-FSDP region is significantly affected by the inclusion of dispersion interactions, the subsequent peaks in the structure factors are indistinguishable between MLIPs and MLIPs+D3. This suggests that the medium-range structural order is somewhat sensitive to the inclusion of dispersion interactions, which plays a key role in reproducing the density and specific features such as the Na-Na and Si-Si partial distributions. The results highlight the importance of considering both short- and medium-range interactions, alongside accurate density reproduction, for reliable modeling of silicate glass structures.

#### Partial Distribution Functions (PDFs)

**Figure 3:**
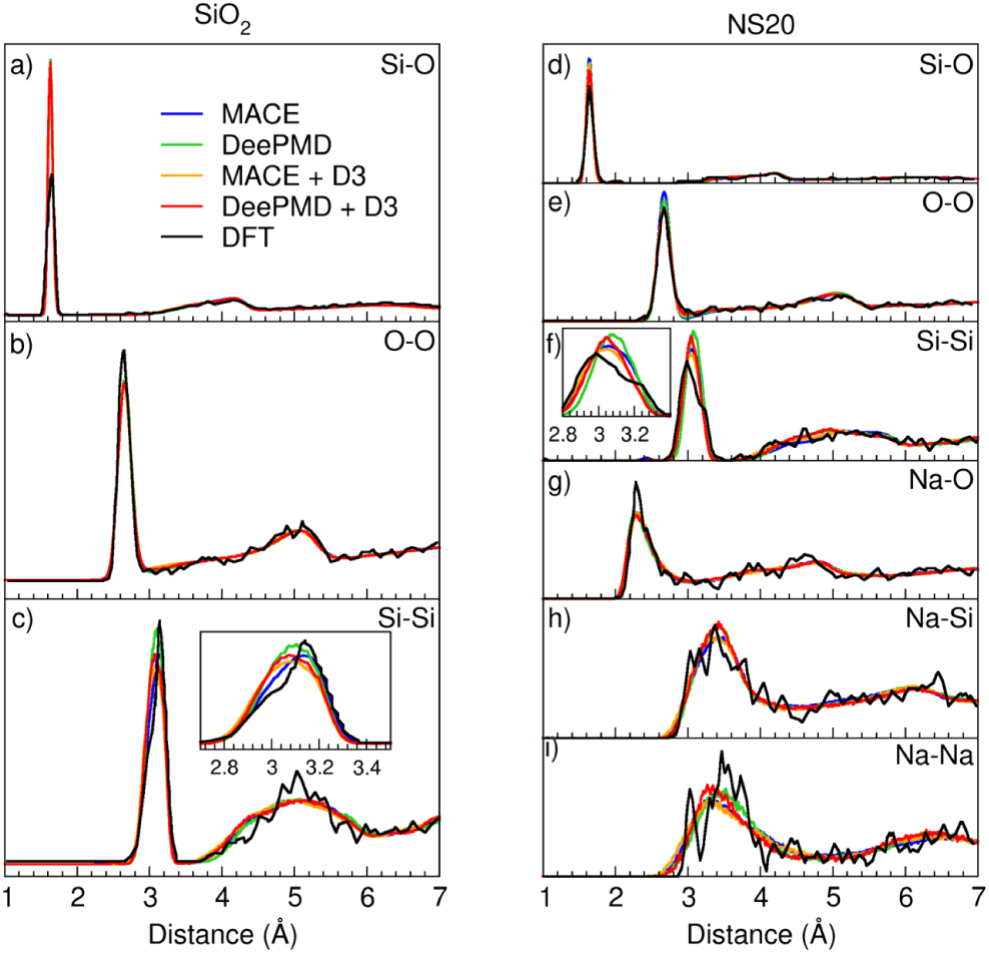
Panels (a)–(c) report the Si-O, O-O, and Si-Si PDFs in SiO_2_ glass. Panels (d)–(i) report the Si-O, O-O, Si-Si, Na-O, Na-Si, and Na-Na PDFs of NS20 glass. All the PDFs have been simulated with the four tested MLIPs and compared to AIMD.

The partial distribution functions of all the element pairs in SiO_2_ and NS20 glasses simulated with MACE and DeePMD with and without D3 dispersion correction are reported in Figure 3 and compared to AIMD data.^14^

Both MACE and DeePMD accurately reproduce all pair distribution functions (PDFs), demonstrating the strong reliability of MLIPs for simulating atomic structures, even with the general MACE model. The inclusion of the D3 dispersion correction has a limited effect on atomic distances, primarily influencing the Si-Si PDF. The first peak of the Si-Si distribution shifts slightly to lower distances, leading to a more compact structure. This improves agreement with DFT data for NS20 but slightly underestimates the Si-Si distance in SiO_2_, likely contributing to the overestimation of silica density when D3 is included. For the DeePMD MLIP, the Na-Na PDF is also affected by dispersion corrections, with the peak shifting to shorter distances, whereas for MACE, this distribution remains largely unchanged.

#### Bond Angle Distribution (BAD)

The reproduction of T-O-T (*T* = glassy network former element) BAD has often been neglected in the development of classical interatomic potentials. In previous papers, we demonstrated the importance of this structural feature for reproducing NMR spectra,^41,42^ silicon polymerization,^11^ and ionic conductivity.^43^

**Figure 4:**
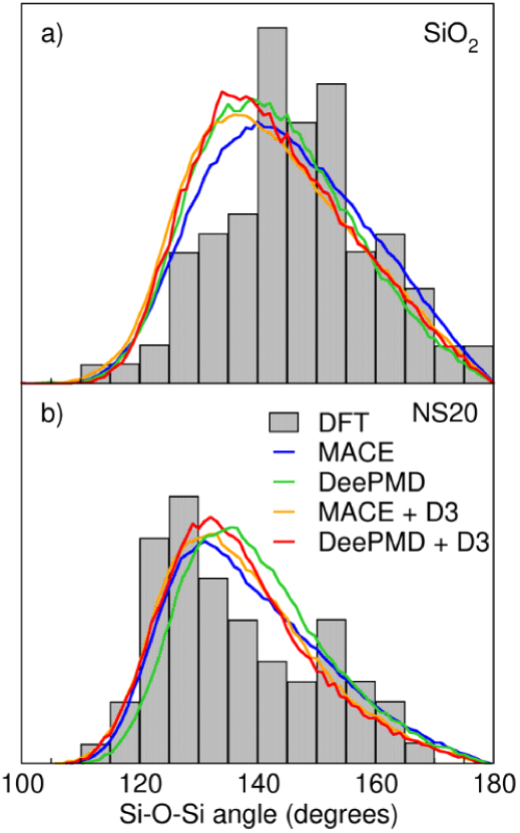
Si-O-Si Bond Angle Distribution (BAD) of SiO_2_ (panel a) and NS20 (panel b) simulated with MACE and DeePMD with and without D3 dispersion correction and compared to AIMD data.

Figure 4 reports the Si-O-Si bond angle distribution of SiO_2_ and NS20 simulated with MACE and DeePMD with and without D3 dispersion correction and compared to AIMD simulations.^14^

Both MACE and DeePMD can reproduce the asymmetric distribution of the Si-O-Si angles in silicate glasses. The inclusion of the dispersion correction improves the Si-O-Si BAD giving more tapered peak shapes centered at lower angles, better reproducing the DFT data. This is coherent with the lower Si-Si distance observed in Figure 3 which can be related to the higher simulated density.

#### Si[Q^n^] Speciation

**Figure 5:**
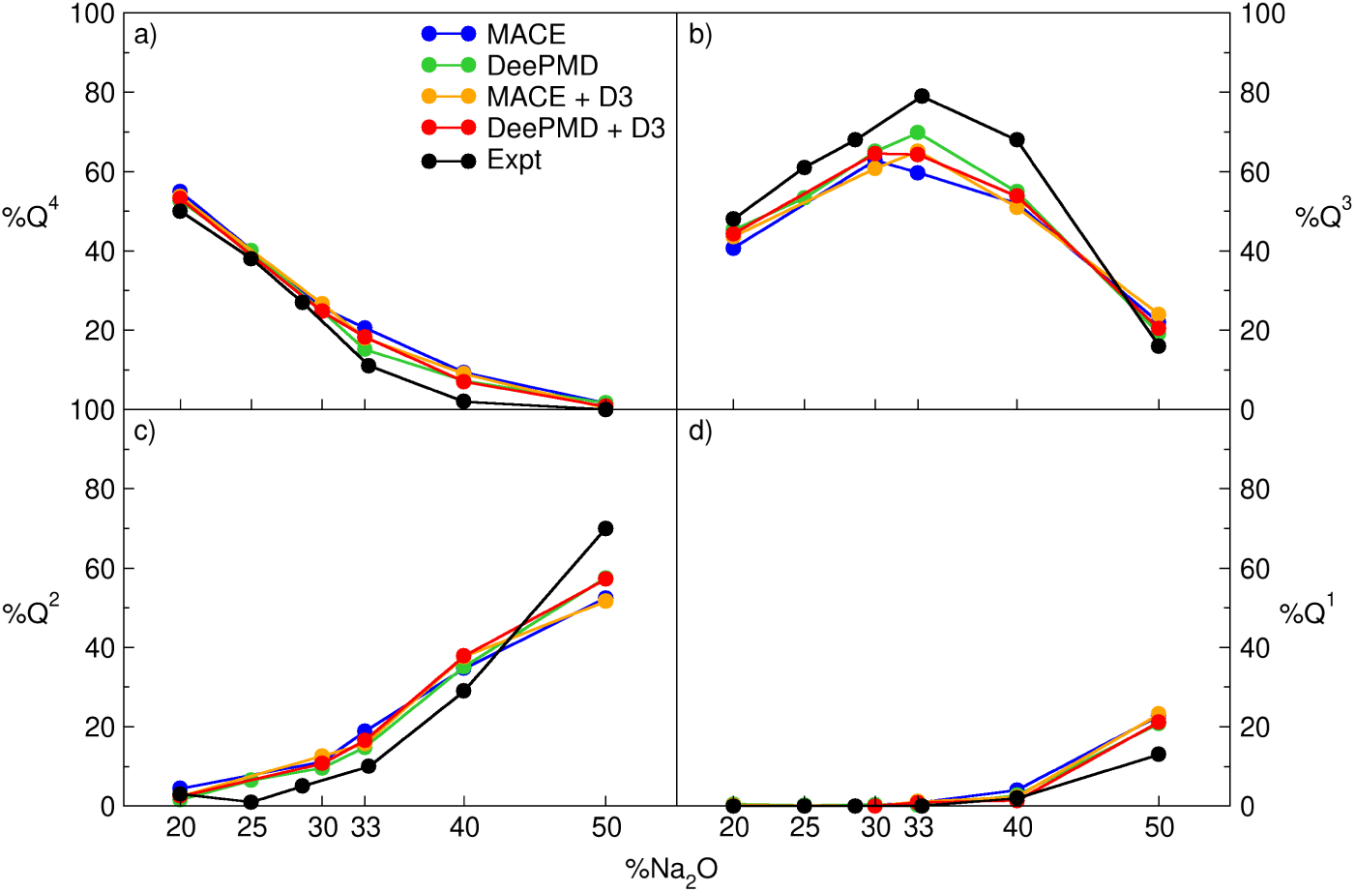
Si[Q^n^] speciation with *n* = 1, 2, 3, and 4 respectively reported in panels (d), (c), (b), and (a), calculated with MACE and DeePMD with and without D3 dispersion correction and compared to experimental data.

The network connectivity in silicate glasses is often expressed as Si[Q^n^] speciation where *Q* stands for quaternary, tetracoordinated species and *n* is the number of bridging oxygens (BO) linked to it. The reproduction of this structural property is fundamental for the correct description of the local atomic structure. Figure 5 reports the Si[Q^n^] percentage speciation as a function of the Na_2_O content simulated with MACE and DeePMD with and without the D3 dispersion correction compared to experimental data^44^ obtained from ^29^Si MAS NMR spectroscopy.

Both MACE and DeePMD accurately reproduce the silicon speciation, with the latter performing slightly better than the former. Despite the shorter simulated Si-Si distance, the inclusion of the D3 dispersion correction does not seem to affect the simulation of the silicon network connectivity.

### Vibrational Density of States

**Figure 6:**
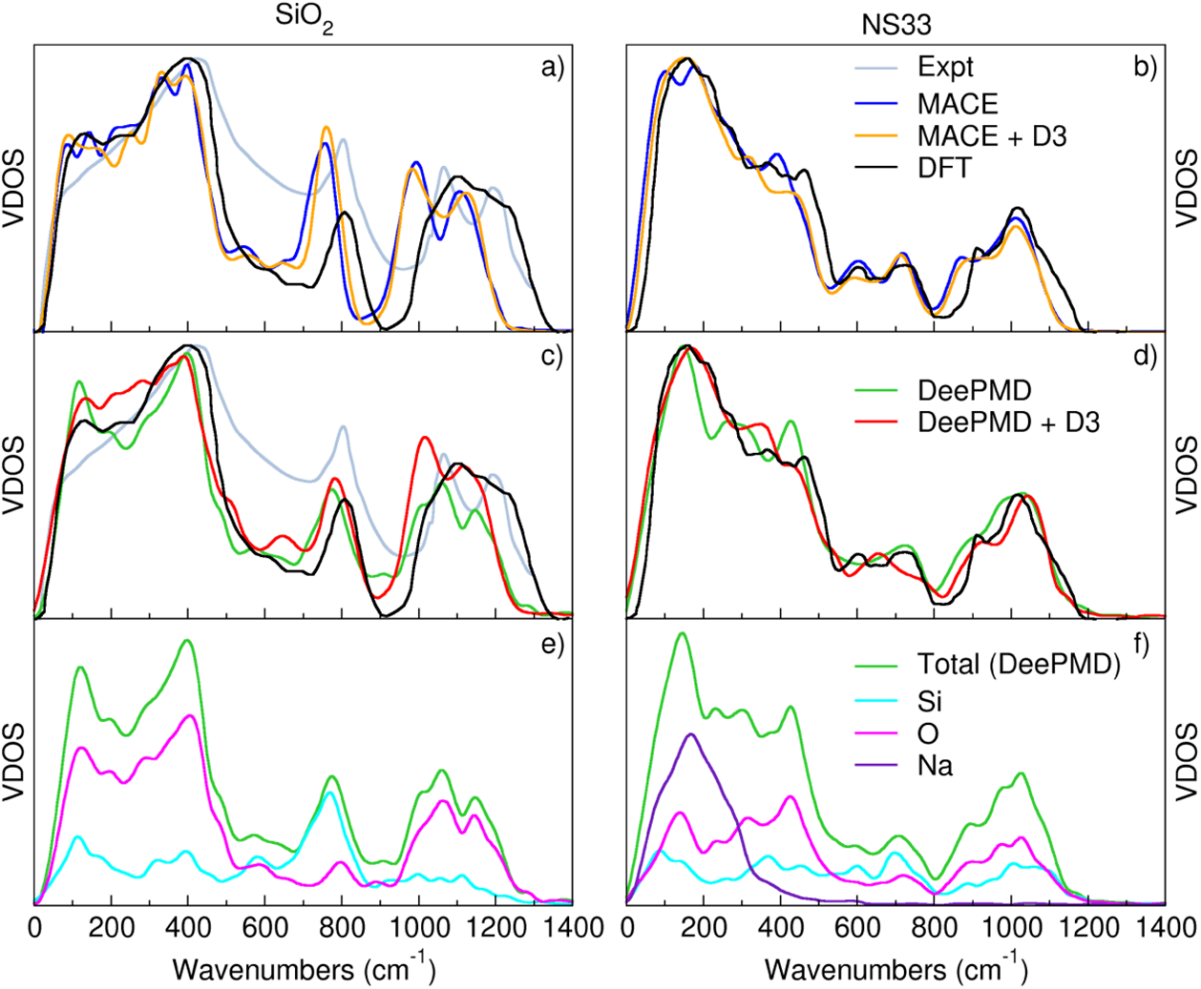
Panels (a) and (b): total VDOS of SiO_2_ and NS33 glasses obtained with MACE with and without D3 correction; (c) and (d) The same VDOS computed with DeePMD and DeePMD-D3 compared to DFT and experimental one (only for SiO_2_). Panels (e) and (f): Partial VDOS of SiO_2_ and NS33 glasses obtained with DeePMD without dispersion correction.

The vibrational density of states (VDOS) provides critical insights into the vibrational dynamics of amorphous materials, reflecting the distribution of vibrational modes over different wavenumbers. In Figure 6, we present the total VDOS for silica glass (panel a) and sodium disilicate glass (panel b), comparing experimental results^45^ (for vitreous silica) with those obtained from density functional theory^46,47^ (DFT), DeePMD, and MACE, both with and without dispersion corrections (D3).

Additionally, the partial VDOS for both glasses, computed at the DeePMD level, are shown in panels (c) and (d) for silica and sodium disilicate, respectively.

The total VDOS of silica glass (Figure 6a–d) exhibits distinct peaks across different wavenumber ranges. According to the interpretation reported in previous works,^22,46,48^ the shoulder between 0 and 200 cm^–1^ corresponds to acoustic modes with contributions from both transverse and longitudinal vibrations, the low-frequency peak at ∼450 cm^–1^ is attributed to rocking motions of oxygen atoms, where they move perpendicular to the Si-O-Si bond planes. These collective motions involve larger-scale network deformations. The midfrequency peak at ∼800 cm^–1^ corresponds to Si-O-Si bond-bending motions. Oxygen atoms vibrate within the Si-O-Si plane, bending the bonds along the bisector of the bond angle. Finally, the high-frequency doublet at 1065 cm^–1^ and 1200 cm^–1^ correspond to the asymmetric T_2_ stretching modes of the SiO_4_ tetrahedra (1065 cm^–1^), where two oxygen atoms move closer to the central silicon while the other two move away whereas the higher peak (∼1200 cm^–1^) is due to A_1_ modes, where all four oxygen atoms move symmetrically in-phase toward the central silicon atom, representing symmetric Si-O stretching.

The vibrational density of states (VDOS) for vitreous silica, computed using DFT,^46^ shows a reasonable overall agreement with the experimental spectrum^45^ (gray curve), particularly in capturing the broad peaks around 450 cm^–1^, 800 cm^–1^, and 1200 cm^–1^. However, the DFT curve (black) underestimates the intensity of the peak near 800 cm^–1^ and fails to fully reproduce the doublet peak at high frequency, presenting it as a single, broad peak rather than the more structured feature observed in the experimental data. This discrepancy suggests that while DFT captures the general vibrational characteristics, it may lack accuracy in describing finer details in the higher wavenumber region.

When comparing MACE (blue), MACE-D3 (orange), DeePMD (green), and DeePMD-D3 (red) with both the DFT and experimental spectra, several key differences emerge. MACE shows a slightly better agreement with the experiment when including the D3 correction (MACE-D3), particularly in reproducing the lower wavenumber features around 450 cm^–1^ and 800 cm^–1^, with better relative intensities than DFT. All the MLIPs deviate from the experimental peak shape at 200 cm^–1^ where MACE-D3 seems to give the best agreement. However, a significant observation is that both MACE and DeePMD successfully capture a splitting of the peak near 1200 cm^–1^, which is consistent with the experimental data and the inclusion of the D3 seems to improve the reproduction of this region. This splitting suggests that both ML potentials capture well the structural complexity of the high-frequency vibrational modes, which are associated with the Si–O stretching vibrations in silica.

Overall, the inclusion of dispersion corrections in MACE-D3 and DeePMD-D3 slightly improves the low- and midfrequency VDOS but also enhances the overall agreement with the experimental spectrum, emphasizing the role of long-range interactions in accurately modeling the vibrational properties of vitreous silica.

The Vibrational Density of States (VDOS) spectra for sodium disilicate glass, calculated using different methods (DFT,^47^ DeePMD, DeePMD-D3, MACE, and MACE-D3), reveal distinctive features due to the presence of sodium ions.

In the low-wavenumber region (0–330 cm^–1^), a prominent peak appears around 150–200 cm^–1^, attributed to the ″rattling″ motions of sodium ions within the disordered glass network. This feature is unique to sodium-containing silicate glasses and is absent in pure silica, underscoring the influence of sodium on the vibrational properties.

In the intermediate-wavenumber region (600–800 cm^–1^), bending modes of the Si-O-Si network are observed, though these modes are broadened due to the network-disrupting effect of sodium. The sodium ions introduce nonbridging oxygens (NBOs) in the glass structure, leading to a more disordered vibrational profile in this range.

At higher wavenumbers (900–1100 cm^–1^), Si-O stretching modes dominate the VDOS. The presence of sodium weakens the Si-NBO bonds, shifting these modes to lower wavenumbers compared to those in pure silica glass. Both MACE and DeePMD capture these trends, aligning reasonably well with the DFT-calculated VDOS, which serves as a reference. Notably, the inclusion of the D3 dispersion correction slightly improves the prediction of the region from 200 to 450 cm^–1^ when applied to DeePMD potential while it does not significantly impact the VDOS calculated with MACE. It is noteworthy that dispersion interactions do not heavily influence these vibrational features in sodium silicate glass and seems to be more important in pure SiO_2_.

### Partial VDOS of Silica Glass

In Figure 6e, the partial VDOS for silica glass, computed using the DeePMD model, provides further insight into the contributions of different atomic species and bonding environments. The low- and intermediate-wavenumber regions (0–850 cm^–1^) are dominated by collective vibrations involving both oxygen and silicon atoms, reflecting the complex bending and deformation modes within the SiO_2_ network. The high-wavenumber region (850–1300 cm^–1^) is primarily associated with oxygen atoms, as the Si-O stretching modes contribute heavily to this region. The ability to resolve these partial contributions helps clarify the vibrational behavior of specific bonds and atoms within the disordered glass matrix.

### Partial VDOS of Sodium Disilicate Glass

The partial VDOS for sodium disilicate glass, also computed using the DeepMD model (Figure 6f), highlights the distinct roles of sodium, silicon, and oxygen atoms in the vibrational spectrum. The low-wavenumber peak (around 150–200 cm^–1^) is dominated by sodium ions, which contribute significantly to the vibrational modes in this region due to their localized rattling motions. Silicon and oxygen atoms dominate the intermediate- and high-wavenumber regions (400–1200 cm^–1^), where Si-O-Si bending and Si-O stretching modes prevail. The inclusion of sodium broadens the VDOS in these regions due to the disruption of the SiO_2_ network structure, as sodium acts as a network modifier. Overall, the comparison of the total VDOS across all models and the partial VDOS for DeepMD (Figure 6) reveals several key insights. Both DeePMD and MACE models qualitatively reproduce the experimental and DFT-calculated VDOS, though the inclusion of dispersion corrections is essential for accurately capturing the high-wavenumber Si-O stretching modes. Additionally, the partial VDOS highlights the importance of sodium in modifying the vibrational landscape of sodium disilicate glass, particularly in the low-wavenumber region, where Na-ion dynamics play a critical role.

### Elastic Properties

The accurate simulation of elastic properties is crucial for the applicability of interatomic potentials in both fundamental research and industrial applications. Elastic moduli, such as the bulk modulus, Young’s modulus, and Poisson’s ratio, are key indicators of a material’s mechanical behavior and its ability to withstand external stresses. Reliable predictions of these properties are essential for designing materials with tailored mechanical performance, particularly in fields such as structural engineering, glass manufacturing, and solid-state electrolytes. In this study, we compute the bulk modulus, which quantifies the resistance of a material to uniform compression, Young’s modulus, which describes its stiffness under uniaxial stress, and Poisson’s ratio, which characterizes the transverse deformation response to an applied axial strain.

**Figure 7:**
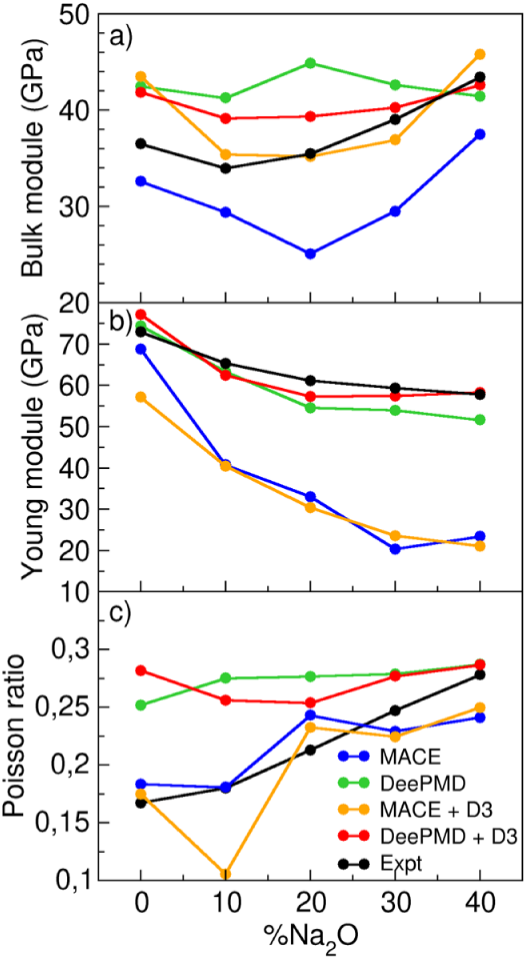
Experimental and simulated Bulk module (a), Young module (b), and Poisson ratio (c).

Figure 7 presents the simulated bulk and Young’s moduli, along with Poisson’s ratio, as a function of Na_2_O content for both MACE and DeePMD models, with and without the D3 dispersion correction, compared against experimental data.^49^

The MACE potential successfully captures the overall trend of bulk and Young’s moduli but systematically underestimates their absolute values, yielding mean absolute errors (MAEs) of 6.85 and 26.12 GPa, respectively. DeePMD, on the other hand, provides a more accurate estimate of Young’s modulus, with an MAE of 4.38 GPa, while its bulk modulus values tend to be slightly overestimated across most compositions. However, DeePMD still achieves better agreement with experiments than MACE, with an MAE of 5.65 GPa for the bulk modulus. In contrast, Poisson’s ratio is more accurately reproduced by MACE, with an MAE of 0.02, closely following the experimental trend. DeePMD tends to overestimate Poisson’s ratio, yielding an MAE of 0.06, with relatively constant values across compositions except for SiO_2_.

The inclusion of the D3 dispersion correction significantly improves the prediction of the bulk modulus, particularly for MACE, reducing its MAE to 2.65 GPa. The corrected values align well with experiments for sodium silicates but still overestimate the bulk modulus of SiO_2_. Similarly, incorporating D3 in DeePMD improves the bulk modulus prediction, reducing its MAE to 3.29 GPa. Interestingly, despite leading to a denser atomic structure, the dispersion correction results in slightly lower bulk modulus values, suggesting a more subtle interplay between dispersion forces and the glass network’s mechanical response.

Young’s modulus is less sensitive to the inclusion of dispersion interactions, with only minor improvements observed at higher Na_2_O content. The MAE for Young’s modulus with D3 correction is 28.77 GPa for MACE and 2.67 GPa for DeePMD. A similar trend is seen for Poisson’s ratio, where D3 slightly lowers the values predicted by both models, yielding MAEs of 0.05 for DeePMD and 0.03 for MACE, except for SiO_2_, where the correction has minimal impact.

## Conclusions

In this work, we evaluated the pretrained MACE-MP-0 MLIP for the first time in the simulation of sodium silicate glasses and compared its performance with a DeePMD-based MLIP specifically trained on these compositions. Additionally, we investigated the impact of explicitly including dispersion interactions using the D3(BJ) correction, implemented in ASE via PyTorch for MACE and in LAMMPS via a custom C++ module for DeePMD.

Our results demonstrate that the pretrained MACE MLIP is a reliable potential for simulating silicate glasses, yielding structural and vibrational properties comparable to those obtained with the DeePMD MLIP. However, DeePMD performs slightly better, particularly in predicting elastic moduli—especially Young’s modulus—and in reproducing Si[Q^n^] speciation and vibrational density of states (VDOS).

The inclusion of D3 dispersion interactions significantly enhances the reproduction of glass density and elastic moduli for both MLIPs, confirming the critical role of dispersion in accurately modeling silicate glasses. Additionally, the VDOS is slightly improved, and structural analyses reveal that dispersion interactions lead to a more compact network by shortening Si-Si and Na-Na distances. However, this improvement comes at the cost of increased computational expense, particularly when using the PyTorch implementation in ASE, where the simulation speed decreases by more than a factor of 4 when including D3. In contrast, our LAMMPS-based C++ implementation for DeePMD maintains high efficiency, with only a minor slowdown (factor of 1.1).

Overall, the pretrained MACE MLIP is a valuable tool for simulating silicate glasses, but its accuracy could be further improved by fine-tuning on glassy structures. Similarly, explicit treatment of dispersion interactions is essential when using MLIPs trained on standard DFT data sets that neglect these effects. Future work should explore the performance of MLIPs trained on ab initio data that inherently include dispersion corrections, providing a more integrated and computationally efficient solution for accurate glass simulations.

## Data Availability

The D3BJ module for LAMMPS and the data that support the findings of this study, including the database and the input files for DeePMD are available from the corresponding author upon reasonable request.

## Supporting Information Available

Theoretical framework, machine learning interatomic potentials (MLIPs), MACE (multi atomic cluster expansion), and Grimme’s D3 empirical correction for dispersion interactions (PDF)
