# Supplementary material for: Revisiting Machine Learning Potentials for Silicate Glasses: The Missing Role of Dispersion Interactions
Source: J Chem Theory Comput. 2025 Apr 24;21(9):4769–78. doi: 10.1021/acs.jctc.5c00218 (PMC12079790; doi:10.1021/acs.jctc.5c00218)
Supplement: Supplementary file 1 — ct5c00218_si_001.pdf [file ct5c00218_si_001.pdf]

SUPPORTING INFORMATION FOR

Revisiting Machine Learning Potentials for Silicate Glasses: The Missing Role of Dispersion Interactions

Alfonso Pedone*, Marco Bertani, and Matilde Benassi

[a] Department of Chemical and Geological Sciences, University of Modena and Reggio Emilia, Modena, 41125, Italy.

Corresponding author: alfonso.pedone@unimore.it

Theoretical Framework

1. Machine Learing Interatomic Potentials (MLIPs)

Two MLIPs frameworks have been evaluated in this work: the Deep Potential MD approach (a variant of the Behler and Parrinello NN Potentials[1]) proposed by Zhang *et al.*;[2,3] and the MACE (Multi Atomic Cluster Expansion) framework developed in Batiata et al.[4] Both models assume that the total potential energy E of the system is the sum of atomic energies E_i associated to each constituting atom *i*. The atomic energies depend in a complex and nonlinear way on the atomic environment of atom *i* (i.e., on nature and disposition of surrounding atoms *j* within a cutoff radius R_c) denoted R^i in equation 1:

$E = \sum_i E(R^i)$	(1)

1.1 DeePMD

In DeePMD The analytical dependence between the atomic energy and the atomic environment is learned by element-specific Fitting Neural Networks ($\mathcal{F}$) with several hidden layers ($\mathcal{L}$) and nodes.

$E_i = \mathcal{F}\big(\boldsymbol{D}\big(\boldsymbol{x}_i,\{\boldsymbol{x}_j\}_{j\in N(i)}\big);\boldsymbol{\theta}_f\big)$	(2)

Where D is the descriptor that depends on degree of freedom of the i-atom, $\boldsymbol{x} = (\boldsymbol{r}_i,\alpha_i)$ with $\boldsymbol{r}_i$ being the Cartesian coordinates and α_i the chemical species. $\boldsymbol{\theta}_f$ represents the parameters of the network. The network can be written as:

$\mathcal{F} = \mathcal{L}^{(out)} \circ \mathcal{L}^{(n)} \circ \mathcal{L}^{(n)} \circ \ldots \circ \mathcal{L}^{(1)}$ $\mathcal{L}^{(out)}(\boldsymbol{d}^{(n)}) = \boldsymbol{W}^{(out)}\boldsymbol{d}^{(n-1)} + b^{out}$ $\boldsymbol{d}^{(n)} = \mathcal{L}^{(n)}(\boldsymbol{d}^{(n-1)}) = tanh(\boldsymbol{W}^{n}\boldsymbol{d}^{(n-1)} + \boldsymbol{b}^{(n)})$ $\boldsymbol{\theta}_f = \{\boldsymbol{W}^{(1)},\boldsymbol{b}^{(1)},\ldots,\boldsymbol{W}^{(n)},\boldsymbol{b}^{(n)},\boldsymbol{W}^{(out)},b^{(out)}\}$	(3)

Where the symbol " ∘ " denotes function composition, $L_i^{(n)}$ is the mapping from layer n − 1 to n. The $\boldsymbol{d}_i^{(n)} \in \mathcal{R}^{M_n}$ denote the values of neurons in layer n and M_n the number of neurons. **W** denotes the weight matrix $\boldsymbol{W}^{(n)} \in \mathcal{R}^{M_n x M_{n-1}}$ and bias vector $\boldsymbol{b}_i^{(n)} \in \mathcal{R}^{M_n}$.

In this work, the DNN used for mapping the energy is composed of two hidden layers of 120 and 60 nodes.

To describe the atomic environment of each atom we used the three-body embedding descriptor called Se_e3[5,6] whose construction is summarized in Figure S1.

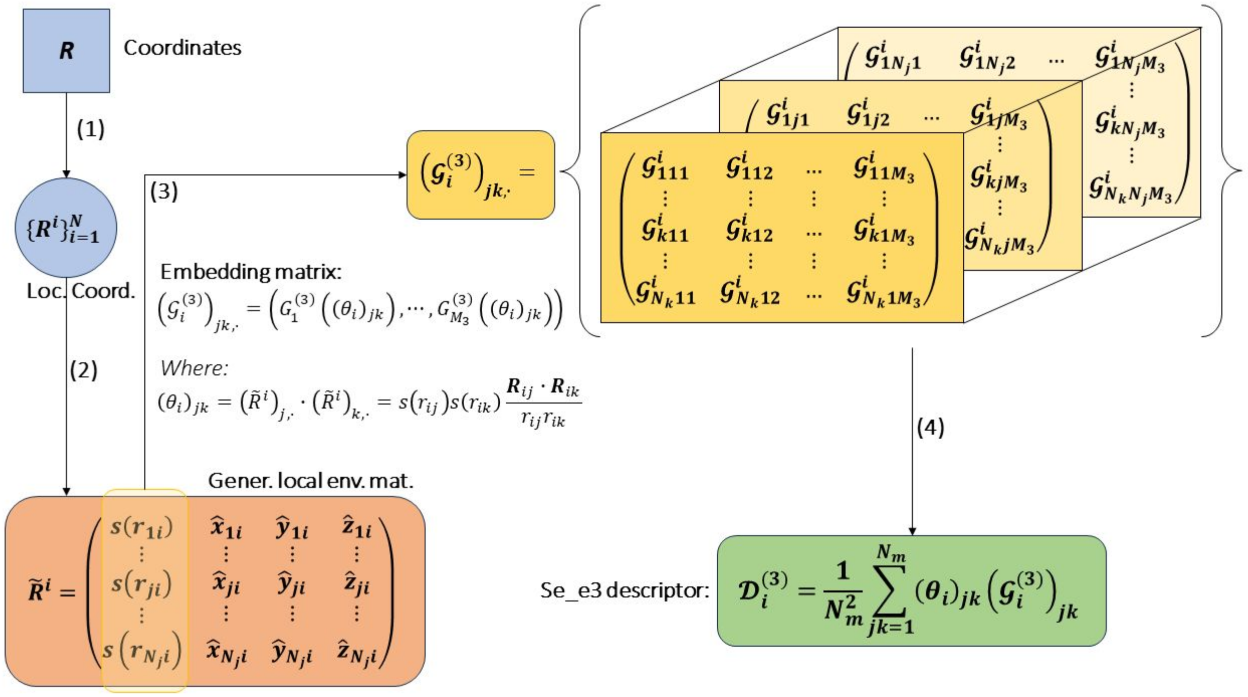

Figure S1: Scheme of the generation of the Se_e3 descriptor implemented in DeePMD.

The descriptor is created starting from the (1) initial transformation of the coordinate matrix $R \in \mathrm{R}^{3,\mathrm{N}}$ of a system containing N atoms, into N local environment matrices $\{R^i\}_{i=1}^{N} \in \mathrm{R}^{3,\mathrm{Nj}}$ containing, for each atom i, the relative coordinate of N_j neighbors within a specified cutoff (r_c = 6 Å) indicated with j index (r_{ji},x_{ji},y_{ji},z_{ji} where $r_{ji} = r_j - r_i$, and x_{ji},y_{ji},z_{ji} are the components of r_{ji}). In step (2), the local coordinates are then mapped into a generalized local environment matrix $\tilde{R}^i \in \mathrm{R}^{4,\mathrm{Nj}}$

$\{x_{ji}, y_{ji}, z_{ji}\} \rightarrow \{s(r_{ji}), \grave{x}_{ji}, \grave{y}_{ji}, \grave{z}_{ji}\}$	(4)

Where $\grave{x}_{ji} = \frac{s(r_{ji})x_{ji}}{r_{ji}}$, $\grave{y}_{ji} = \frac{s(r_{ji})y_{ji}}{r_{ji}}$, and $\grave{z}_{ji} = \frac{s(r_{ji})z_{ji}}{r_{ji}}$ while $s(r_{ji})$ is a function that reduces the weight of atoms that are farther from the central atom, smoothing it to zero at the cutoff, defined as:

$s(r_{ji}) = \begin{cases} \frac{1}{r_{ji}}, r_{ji} < r_{cs} \\ \frac{1}{r_{ji}}\left\{\left(\frac{r_{ji} - r_{cs}}{r_c - r_{cs}}\right)^3 \left(-6\left(\frac{r_{ji} - r_{cs}}{r_c - r_{cs}}\right)^2 + 15\frac{r_{ji} - r_{cs}}{r_c - r_{cs}} - 10\right) + 1\right\}, r_{cs} \leq r_{ji} \leq r_c \\ 0, r_{ji} > r_c \end{cases}$	(5)

Here r_c is the spherical cutoff and r_{cs} is a smooth cutoff parameter. These were set to 7.0 and 6.5 Å, respectively.

In step (3) $s(r_{ij})$ is mapped to an embedding tensor $\left(\mathcal{G}_i^{(3)}\right)_{jk}$ through an embedding neural network that incorporates the angles between atoms:

$\left(\mathcal{G}_i^{(3)}\right)_{jk,\cdot} = \left(G_1^{(3)}\left((\theta_i)_{jk}\right), \cdots, G_{M_3}^{(3)}\left((\theta_i)_{jk}\right)\right)$	(6)

Where $G^{(3)}$ indicates a vector of length M_3 (an arbitrary value decided by the operator) obtained from a trainable deep neural network (DNN).

In equation 4, the value of $(\theta_i)_{jk}$, which changes with the angle between atoms, is obtained as:

$(\theta_i)_{jk} = \left(\tilde{R}_i\right)_{j,\cdot} \cdot \left(\tilde{R}_i\right)_{k,\cdot} = s(r_{ij})s(r_{ik})\frac{R_{ij} \cdot R_{ik}}{r_{ij}r_{ik}}$	(7)

In step (4), the Se_e3 descriptor is finally defined as:

$D_i^{(3)} = \frac{1}{N_m^2}\sum_{jk=1}^{N_m} (\theta_i)_{jk}\left(\mathcal{G}_i^{(3)}\right)_{jk}$	(8)

We refer to the original work in references[5,6] for further details about the mathematical properties of this descriptor.

1.2 MACE (Multi Atomic Cluster Expansion)

MACE builds upon the Message Passing Neural Network (MPNN) framework to compute atomic energies.[4,7,8] Atomic positions and chemical elements are represented as graph nodes, with edges defined by atomic interactions within a cutoff radius. N(i) is the set of neighbours of atom i within the cutoff radius.

Each atom i is characterized by a state $\sigma_i^{(t)} = \left(\boldsymbol{r}_i, \boldsymbol{\theta}_i, \boldsymbol{h}_i^{(t)}\right)$ where $\boldsymbol{r}_i$ denotes the atom's Cartesian position vector, $\boldsymbol{\theta}_i$ a set of its fixed attributes such as the chemical element represented by a one-hot embedding and $\boldsymbol{h}_i^{(t)}$ its learnable features. The superscript denotes the iteration step in the message-passing process, where the atomic features are updated progressively through multiple iterations.

The network updates atomic features through message passing, where messages $m_i^{(t)}$ are constructed by pooling over neighboring atoms j ($\bigoplus_{j \in N(i)}$), using a learnable message function M_t

$m_i^{(t)} = \bigoplus_{j \in N(i)} M_t\left(\sigma_i^t, \sigma_j^t\right)$	(9)

and updating the features via a learnable update function U_t

$h_i^{(t+1)} = U_t\left(\sigma_i^t, m_i^t\right)$	(10)

After T message construction and update steps, a learnable readout function R_t map the node states $\sigma_i^{(t)}$ to the site energy of atom i:

$E_i = \sum_{\boldsymbol{t}} R_t\left(\sigma_i^{(t)}\right)$	(11)

MACE extends this framework by leveraging the Atomic Cluster Expansion descriptors to construct equivariant messages with high-body-order interactions.[9]

The state of an atom i is represented by an array of features $\mathbf{h}_i$ which is expressed in a radial and spherical hamonic basis, and thus its elements are always indexed by l and m.

At the beginning of the process, the node features $\boldsymbol{h}_i^{(0)}$ are inizialized as a learnable one hot embedding (if there are S different atomic species, each species is represented as an S-dimensional vector with a single **1** in the position corresponding to its species and **0** everywhere else) of the chemical elements with atomic number z_i into k learnable channels:

$$h_{ik,00}^{(0)} = \sum_z W_{kz} \delta_{zz_i} \quad (12)$$

This transforms the categorical atomic identity into a continuous vector representation, allowing the model to learn chemical similarities and differences more efficiently, leading to some transferability between molecules with different elements.[8]

The zeros subscripts lm correspond to scalar initial features (rotational invariant). The higher order elements of $\boldsymbol{h}_i^{(0)}$ with non zero lm indices are implicitly initialized to zero. At the beginning of each iteration, the node features are linearly mixed together resulting in the feature vector $\tilde{\boldsymbol{h}}_j$.

$$\tilde{h}_{j,kl_2m_2}^{(t)} = \sum_{\tilde{k}} W_{k\tilde{k}l_2}^{(t)} h_{i\tilde{k}l_2m_2}^{(t)} \quad (13)$$

Next, the features of each of the neighbouring atoms (the edges in the graph) are embedded (combined) using a learnable radial basis $R_{kl_1l_2l_3}^{(t)}(r_{ij})$ constructed using spherical Bessel functions j_0^n (r_{ij}):

$$R_{kl_1l_2l_3}^{(t)}(r_{ij}) = MLP\left(\left\{j_0^n(r_{ij})\right\}_n\right) \quad (14)$$

a set of spherical harmonics $Y_{l_1}^{m_1}(\boldsymbol{r}_{ij})$ and the learnable embedding of the previous node features $\tilde{h}_{j,kl_2m_2}^{(t)}$. The atomic basis $\boldsymbol{A}_i^{(t)}$ are obtained by pooling over the neighbours N(i) to obtain

permutational invariant 2-body features maintaining full directional information, and thus full information of the atomic environment:

$A^{(t)}_{i,kl_3m_3} = \sum_{l_1m_1,l_2m_2} C^{l_3m_3}_{l_1m_1,l_2m_2} \sum_{j\in N(i)} R^{(t)}_{kl_1l_2l_3}(r_{ij}) Y^{m_1}_{l_1}(\boldsymbol{r}_{ij}) \tilde{h}^{(t)}_{j,kl_2m_2}$	(15)

Where $C^{l_3m_3}_{l_1m_1,l_2m_2}$ are the standard Clebsh-Gordon coefficient ensuring that $A^{(t)}_{i,kl_3m_3}$ keep the correct equivariance, r_{ij} is the scalar interatomic distance, and $\boldsymbol{r}_{ij}$ the corresponding unit vector.

Multi-body features are constructed from the pair-wise atomic basis $\boldsymbol{A}^{(t)}_i$ features by performing tensor products of the features and symmetrizing:

$\boldsymbol{B}^{(t)}_{i,\eta_v,kLM} = \sum_{\boldsymbol{lm}} C^{LM}_{\eta_v,\boldsymbol{lm}} \prod_{\xi=1}^{v} \sum_{\tilde{k}} W^{(t)}_{k\tilde{k}l_\xi} A^{(t)}_{i,kl_\xi m_\xi} \qquad \boldsymbol{lm} = (l_1m_1,\ldots,l_vm_v)$	(16)

Where the coupling coefficients $C^{LM}_{\eta_v,\boldsymbol{lm}}$ corresponding to the generalized Clebsh-Gordan coefficients ensure that $\boldsymbol{B}^{(t)}_{i,\eta_v,kLM}$ are L-equivariant, the weights $W^{(t)}_{k\tilde{k}l_\xi}$ mix the channels (k) of $\boldsymbol{A}^{(t)}_i$, and v is a given correlation order. The additional index η_v enumerates all possible couplings of $l_1,\ldots,l_v$ features that yield the selected equivariance specified by the L index. Therefore, the $B^{(t)}_i$ features are constructed up to some maximum v identifying the order of the many-body expansion.

Finally, the message $\boldsymbol{m}^{(t)}_i$ on each atom is formed as a linear expansion of the symmetrized many body features of the neighbours:

$m^{(t)}_{i,kLM} = \sum_{v} \sum_{\eta_v} W^{(t)}_{z_i\eta_v,kL} \boldsymbol{B}^{(t)}_{i,\eta_v,kLM}$	(17)

Where $W^{(t)}_{z_i\eta_v,kL}$ is a learnable weight matrix that depend on the chemical element of the receiving atom and message symmetry L.

In MACE, the update stage is a linear function of the message and the features of previous iteration:

$$h_{i,kLM}^{(t+1)} = \sum_{\tilde{k}} W_{kL,\tilde{k}}^{(t)} m_{i,\tilde{k}LM}^{(t)} + \sum_{\tilde{k}} W_{z_i kL,\tilde{k}}^{(t)} h_{i,\tilde{k}LM}^{(t)} \quad (18)$$

Finally, in the readout phase, the invariant part of the node features is mapped to a hierarchical decomposition of site energies via readout function:

$$E_i = E_i^{(0)} + E_i^{(1)} + \ldots + E_i^{(T)}, \text{ where}$$

$$E_i^{(t)} = \sum_{\boldsymbol{t}} R_t\left(\boldsymbol{h}_i^{(t)}\right) = \begin{cases} \sum_{\tilde{\boldsymbol{k}}} W_{readout,\tilde{k}}^{(t)} h_{ik,00}^{(t)} & if\ t < T \\ MLP_{readout}^{(t)}\left(\left\{h_{ik,00}^{(t)}\right\}\right), & t = T \end{cases} \quad (19)$$

The key model size control in MACE are the number of embedding channels k and the highest order L_{max} of the symmetric features $\boldsymbol{B}_{i,\eta_v,kLM}^{(t)}$.

2. Grimme's D3 Empirical Corretion for Dispersion Interactions

The D3 dispersion correction method by Grimme is given by the sum of two and three-body energies.[10,11] In this work we have implemented in LAMMPS only the most important two body term with the the Becke-Johnson (BJ) damping function,[12] is applied to avoid singularities at short distances:

$$E_{D3(BJ)}^{(\mathbf{2})} = -\sum_{AB} s_6 \frac{C_6^{AB}}{r_{AB}^6 + \left(a_1 R_0^{AB} + a_2\right)^6} + s_8 \frac{C_8^{AB}}{r_{AB}^8 + \left(a_1 R_0^{AB} + a_2\right)^8} \quad (20)$$

Where the scaling factor s_6=1 for standard Density Functionals Approximations to ensure the right long-range asymptotics where only the C_6 term is relevant. The a_1, a_2 and s_8 are functional dependent parameters fitted to noncovalent interaction energies.

R_0^{AB} is a fixed cutoff radius for atom pairs AB computed from first-principles. The dispersion coefficients C_6^{AB} depend on the local coordination environment of each atom. They are interpolated from reference values computed using time-dependent density functional theory (TDDFT) for

different molecular environments, ensuring a smooth transition between different atomic environments. The interpolation follows:

$$C_6^{AB}(CN_A,CN_B) = \frac{Z}{W}$$	(21)
$$Z = \sum_{i}^{N_A}\sum_{j}^{N_B} C_{6,ref}^{AB}\left(CN_i^A,CN_j^B\right)L_{ij}$$	(22)
$$W = \sum_{i}^{N_A}\sum_{j}^{N_B} L_{ij}$$	(23)
$$L_{ij} = e^{-k_3\left[\left(CN^A - CN_i^A\right)^2 + \left(CN^B - CN_j^B\right)^2\right]}$$	(24)
$$CN^A = \sum_{B\neq A}^{N_{at}} \frac{1}{1 + e^{-k_1\left(\frac{k_2(R_{A,cov}+R_{B,cov})}{r_{AB}} - 1\right)}}$$	(25)

Where N_A and N_B are number of supporting points for atoms A and B, respectively. CN^A and CN^B are coordination numbers for the atom pair AB in the system of interest, and CN_i^A and CN_j^B are those for the two reference systems I and j, for which $C_{6,ref}^{AB}\left(CN_i^A,CN_j^B\right)$ is the precomputed value. The parameters k1, k2 and k3 are equal to 16, 4/3 and 4, respectively. $R_{A/B,\mathrm{cov}}$ are a scaled covalent single-bond radii of atoms *A* and B taken from Pyykkö and Atsumi.[13]

The coefficient C_8 is computed from C_6 by

$$C_8^{AB} = 3k_{AB}C_8^{AB}$$	(26)

Where k_{AB} is an atom pair dependent coefficient

$$k_{AB} = s_{42}\sqrt{\sqrt{Z_A}\left(\frac{\langle r^4\rangle^A}{\langle r^2\rangle^A}\right)\sqrt{Z_B}\left(\frac{\langle r^4\rangle^B}{\langle r^2\rangle^B}\right)}$$	(27)

s_{42} is an empirical parameter equal to ½. $\langle r^4 \rangle^A$ and $\langle r^2 \rangle^A$ are quadrupole and dipole moment expectation values, respectively, computed for the free atom A. Their ratio is scaled with an scaling term which depend on the nuclear charge of the atom A.

In our LAMMPS module all the parameters are included at the exception of the parameters s_6 , s_8 , a_1 and a_2 parameters that must be provided together with the cutoff radius for short range interactions (10Å).

These are the commands that must be included in the LAMMPS input file

```
# potential
pair_style     hybrid/overlay deepmd NS_deepmd.pb D3BJ 10.0 1.0 0.7875 0.4289 4.4407
pair_coeff     * * deepmd
pair_coeff     * * D3BJ
```

In our case (PBE functional), s_6 =1, s_8 =0.7875, a_1 =0.4289 and a_2 =4.4407.

References

(1) Behler, J.; Parrinello, M. Generalized Neural-Network Representation of High-Dimensional Potential-Energy Surfaces. *Phys. Rev. Lett.* **2007**, *98* (14), 146401. https://doi.org/10.1103/PhysRevLett.98.146401.

(2) Wang, H.; Zhang, L.; Han, J.; E, W. DeePMD-Kit: A Deep Learning Package for Many-Body Potential Energy Representation and Molecular Dynamics. *Comput. Phys. Commun.* **2018**, *228*, 178–184. https://doi.org/10.1016/j.cpc.2018.03.016.

(3) Zhang, L.; Han, J.; Wang, H.; Car, R.; E, W. Deep Potential Molecular Dynamics: A Scalable Model with the Accuracy of Quantum Mechanics. *Phys. Rev. Lett.* **2018**, *120* (14), 143001. https://doi.org/10.1103/PhysRevLett.120.143001.

(4) Batatia, I.; Kovács, D. P.; Simm, G. N. C.; Ortner, C.; Csányi, G. MACE: Higher Order Equivariant Message Passing Neural Networks for Fast and Accurate Force Fields. arXiv January 26, 2023. https://doi.org/10.48550/arXiv.2206.07697.

(5) Wang, X.; Wang, Y.; Zhang, L.; Dai, F.; Wang, H. A Tungsten Deep Neural-Network Potential for Simulating Mechanical Property Degradation under Fusion Service Environment. *Nucl. Fusion* **2022**, *62* (12), 126013. https://doi.org/10.1088/1741-4326/ac888b.

(6) Zeng, J.; Zhang, D.; Lu, D.; Mo, P.; Li, Z.; Chen, Y.; Rynik, M.; Huang, L.; Li, Z.; Shi, S.; Wang, Y.; Ye, H.; Tuo, P.; Yang, J.; Ding, Y.; Li, Y.; Tisi, D.; Zeng, Q.; Bao, H.; Xia, Y.; Huang, J.; Muraoka, K.; Wang, Y.; Chang, J.; Yuan, F.; Bore, S. L.; Cai, C.; Lin, Y.; Wang, B.; Xu, J.; Zhu, J.-X.; Luo, C.; Zhang, Y.; Goodall, R. E. A.; Liang, W.; Singh, A. K.; Yao, S.; Zhang, J.; Wentzcovitch, R.; Han, J.; Liu, J.; Jia, W.; York, D. M.; E, W.; Car, R.; Zhang, L.; Wang, H. DeePMD-Kit v2: A Software Package for Deep Potential Models. arXiv April 18, 2023. https://doi.org/10.48550/arXiv.2304.09409.

(7) Batatia, I.; Benner, P.; Chiang, Y.; Elena, A. M.; Kovács, D. P.; Riebesell, J.; Advincula, X. R.; Asta, M.; Avaylon, M.; Baldwin, W. J.; Berger, F.; Bernstein, N.; Bhowmik, A.; Blau, S. M.; Cărare, V.; Darby, J. P.; De, S.; Della Pia, F.; Deringer, V. L.; Elijošius, R.; El-Machachi, Z.; Falcioni, F.; Fako, E.; Ferrari, A. C.; Genreith-Schriever, A.; George, J.; Goodall, R. E. A.; Grey, C. P.; Grigorev, P.; Han, S.; Handley, W.; Heenen, H. H.; Hermansson, K.; Holm, C.; Jaafar, J.; Hofmann, S.; Jakob, K. S.; Jung, H.; Kapil, V.; Kaplan, A. D.; Karimitari, N.; Kermode, J. R.; Kroupa, N.; Kullgren, J.; Kuner, M. C.; Kuryla, D.; Liepuoniute, G.; Margraf, J. T.; Magdău, I.-B.; Michaelides, A.; Moore, J. H.; Naik, A. A.; Niblett, S. P.; Norwood, S. W.; O'Neill, N.; Ortner, C.; Persson, K. A.; Reuter, K.; Rosen, A. S.; Schaaf, L. L.; Schran, C.; Shi, B. X.; Sivonxay, E.; Stenczel, T. K.; Svahn, V.; Sutton, C.; Swinburne, T. D.; Tilly, J.; van der Oord,

C.; Varga-Umbrich, E.; Vegge, T.; Vondrák, M.; Wang, Y.; Witt, W. C.; Zills, F.; Csányi, G. A Foundation Model for Atomistic Materials Chemistry. arXiv March 1, 2024. https://doi.org/10.48550/arXiv.2401.00096.

(8) Batatia, I.; Batzner, S.; Kovács, D. P.; Musaelian, A.; Simm, G. N. C.; Drautz, R.; Ortner, C.; Kozinsky, B.; Csányi, G. The Design Space of E(3)-Equivariant Atom-Centered Interatomic Potentials. arXiv November 24, 2022. https://doi.org/10.48550/arXiv.2205.06643.

(9) Ortner, C. On the Atomic Cluster Expansion: Interatomic Potentials and Beyond. 2023. https://doi.org/10.25950/c7f24234.

(10) Grimme, S.; Antony, J.; Ehrlich, S.; Krieg, H. A Consistent and Accurate Ab Initio Parametrization of Density Functional Dispersion Correction (DFT-D) for the 94 Elements H-Pu. *J. Chem. Phys.* **2010**, *132* (15), 154104. https://doi.org/10.1063/1.3382344.

(11) Grimme, S.; Hansen, A.; Brandenburg, J. G.; Bannwarth, C. Dispersion-Corrected Mean-Field Electronic Structure Methods. *Chem. Rev.* **2016**, *116* (9), 5105–5154. https://doi.org/10.1021/acs.chemrev.5b00533.

(12) Johnson, E. R.; Becke, A. D. A Post-Hartree-Fock Model of Intermolecular Interactions: Inclusion of Higher-Order Corrections. *J. Chem. Phys.* **2006**, *124* (17), 174104. https://doi.org/10.1063/1.2190220.

(13) Pyykkö, P.; Atsumi, M. Molecular Single-Bond Covalent Radii for Elements 1–118. *Chem. – Eur. J.* **2009**, *15* (1), 186–197. https://doi.org/10.1002/chem.200800987.
